# A Hospital Matron on Hospital Morals

**Published:** 1886-12-25

**Authors:** 


### A Hospital Matron on Hospital Morals.

We have received the following letters from the Council of the Hospitals Association :—

The Hospitals Association,
Norfolk House, Norfolk Street,
London, W.C., Dec. 17, 1886.

Dear Sir,—I was requested by the Council, at their meeting held on the 15th inst., to forward you the enclosed letter, received from Miss Wood, Lady Superintendent, Hospital for Sick Children, Great Ormond Street, W.C.

Yours faithfully,
Duncan J. Caddy, Assist. Sec.

To the Editor of The Hospital.

---

The Hospital for Sick Children,
Great Ormond Street, Bloomsbury.
Dec. 10, 1886.

Dear Sir,—I shall feel obliged by your not sending me any more numbers of The Hospital. This last one I return. I am so grieved to see in it the story "Sister Olive," conveying quite a false idea of the discipline and arrangements of hospital life, and, moreover, lowering the whole tone of moral uprightness in the workers in those institutions. I should be ashamed to see such a publication lying on my table. I think that it would be well if this letter were laid before the Council.

I am, Sir, yours faithfully,
C. J. Wood, Lady Supt.

The Assistant Secretary.

To our Readers.

Concerning Miss Wood's letter, we cannot help feeling that the "virtuous indignation" is a little too elaborately overdone. But our readers shall judge for themselves. In the story entitled "Sister Olive, a Hospital Romance," Sister Olive, a lady of character and education, is in charge of a ward in a County Infirmary. Dr. Grant, a house-physician who has been qualified five years, and is a man of unblemished reputation, has the misfortune to esteem Sister Olive very highly, and to wish to make her his wife. He is about to leave the infirmary to enter upon private practice, and, after much self-communing, decides to tell Sister Olive of his sincere affection, and of his hope that she may one day become the lifelong companion of his home. Having no other place in which he could conveniently speak to her before his departure, he asks her, at a given hour of the afternoon, to speak to him in the house-physician's room. That is all. That is the scene which is "lowering the whole tone of moral uprightness in the workers in those institutions" (hospitals). Because this has appeared in the The Hospital, Miss Wood would "be ashamed to see such a publication lying on her table." There is one answer to such fantastic prudery—"*Honi soit qui mal y pense.*" Those of our readers who are familiar with the interior of a county infirmary know that the single house-physician or surgeon occupies an unique position. He must be a man of irreproachable character in order to obtain the appointment. He must preserve his character in order to retain his position. He is received as a friend into the families of the Honorary Staff; and is, in fact, a man necessarily as much above suspicion as Cæsar's wife. For such a man to ask an educated woman, on the eve of his final departure from the hospital, to speak to him in his sitting-room, because it is the only available place in which he could possibly speak to her privately, is not only no offence against propriety or good taste, but it could never be even thought of in such a connection, except by one who is determined to put an evil construction upon the most innocent acts, if such a construction be within the range of the remotest possibility. We repeat, "Let evil be to him that evil thinks."

A Recent Review.

Whilst offering this explanation, we take the opportunity of apologising to Miss Wood for an inadvertence which occurred in a recent number of The Hospital. Our reviewer, in dealing with Miss Wood's book on "Nursing,"* etc., spoke of her as *Mrs. Wood.* It was of course a mistake easily made, as he had no knowledge of Miss Wood even by name. It was unfortunate, also, that he felt bound to make some rather severe strictures upon the publication in question—more drastic, indeed, than the editor could fully approve; and the strictures were therefore toned down before the review appeared in The Hospital. If Miss Wood felt aggrieved at the strictures, or the mistake about herself, we hope she will consider this a sufficient explanation.—Ed. T. H.

---

* See The Hospital for Oct. 30, p. 82.

